# Emerging role of m6A methylation modification in ovarian cancer

**DOI:** 10.1186/s12935-021-02371-3

**Published:** 2021-12-11

**Authors:** Lin-Lin Chang, Xia-Qing Xu, Xue-Ling Liu, Qian-Qian Guo, Yan-Nan Fan, Bao-Xia He, Wen-Zhou Zhang

**Affiliations:** 1 Department of Pharmacy, Affiliated Tumour Hospital of Zhengzhou University, Henan Cancer Hospital, 127# Dongming Rd, Zhengzhou, 450008 Henan China; 2 Department of Clinical Pharmacy, Zhengzhou Central Hospital Affiliated To Zhengzhou University, Zhengzhou, China

**Keywords:** m6A modification, Methyltransferase, Demethylase, RNA binding protein, OC

## Abstract

m6A (N6-methyladenosine) methylation, a well-known modification in tumour epigenetics, dynamically and reversibly fine tunes the entire process of RNA metabolism. Aberrant levels of m6A and its regulators, which can predict the survival and outcomes of cancer patients, are involved in tumorigenesis, metastasis and resistance. Ovarian cancer (OC) ranks first among gynaecological tumours in the causes of death. At first diagnosis, patients with OC are usually at advanced stages owing to a lack of early biomarkers and effective targets. After treatment, patients with OC often develop drug resistance. This article reviews the recent experimental advances in understanding the role of m6A modification in OC, raising the possibility to treat m6A modification and its regulators as promising diagnostic markers and therapeutic targets for OC.

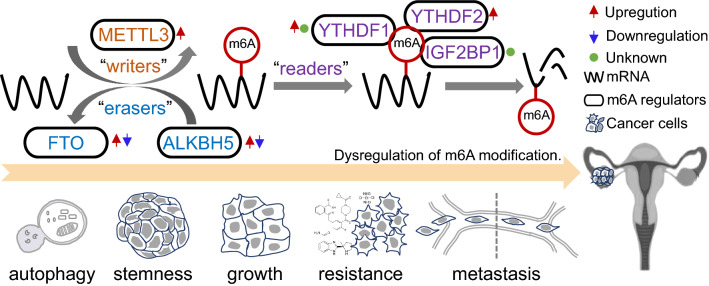

## Background

Ovarian cancer (OC) is the leading death among gynaecological tumours, attracting the attention of researchers [1–3]. According to its histopathology, OC consists of sex cord-stromal tumours, germ cell tumours and epithelial ovarian cancer (EOC), the last of which accounts for 85% of OC cases and is associated with an unimproved 5-year survival rate (40%) [4]. Furthermore, 90% of EOCs are identified as serous ovarian cancers, which exhibit a poor 10-year survival rate (26%) [5–7].

One of the hot research topics in OC is early detection and prediction. In addition to imaging, liver function and complete blood count, tumour markers [usually cancer antigen 125 (CA-125)] can assist in primary diagnosis and progression evaluation, and their assessment is minimally invasive [8, 9]. A concentration of 22 U/mL CA-125 can detect 66.5% (95% CI 49.5–58.4) of ovarian cancers, and an increase in the cut-off to 35 U/mL allows detection of 41.3% (95% CI 33.5–49.5) [8, 9]. However, the concentration of serum CA-125 is controversial because fixed CA-125 cut-offs show no mortality benefit in the general population [9]. Moreover, the AURELIA trial and MRCOV05 trial results indicated that CA-125 was not suitable for surveillance, shown by the lack of an obvious correlation with survival [10]. Thus, there is a lack of efficient detective biomarkers, contributing to  ~ 80% OC patients diagnosed at metastatic advanced-stage, most of which will develop resistance to current clinical therapies [11, 12]. For advanced OC patients, the first-line treatment usually is surgical cytoreduction and subsequent adjuvant chemotherapy (paclitaxel and carboplatin) and then maintenance therapy [inhibitors of poly (ADP-ribose) polymerase (PARPis), bevacizumab or their combination] [13]. Although the paclitaxel and platinum regimen has been the standard adjuvant chemotherapy over the last 30 years, nearly all patients will endure relapse within 2 years, despite achieving complete remission [10, 13]. PARPis as maintenance monotherapy in SOLO-1 trials displayed promising outcomes for patients with *BRCA* mutation and/or genomic instability, termed as homologous recombination deficiency (HRD) [14, 15]*.* Niraparib also showed a survival benefit (vs. placebo) for the overall population in the PRIMA trial, extending patient survival regardless of *BRCA* mutation and indicating other possible targets for OC treatment [16]. Cumulative studies have mainly focused on exploring the front-line therapy regimen, neoadjuvant systemic therapy and maintenance therapy, whereas clinical trials have brought no significant improvement in overall survival for OC patients [17]. Referring to immunotherapy, checkpoint inhibitors have poor efficacy because most OCs are characterized by low levels of neoantigens on cancer cells [17]. In summary, OC is still incurable [17–19]. Therefore, a greater understanding of the signalling cascades involved in OC progression to identify markers for surveillance detection and targets for new therapies will aid in diagnosis and treatment, which are vital to clinical outcomes for OC patients.

N6-methyladenosine (m6A), a posttranscriptional modification in RNA, is dynamical and reversible [20, 21]. It was discovered in the 1970s, providing a potential avenue for epigenetic studies and a focus for disease-related researches [22–24]. Aberrant m6A levels are observed in many pathological processes, including spermatogenesis, adipogenesis, heat shock response, circadian rhythm and T cell homeostasis [24]. During porcine spermatogenesis, m6A in transcripts mediates SET domain bifurcated histone lysine methyltransferase 1 (*SETDB1*) and forkhead box O3 (*FOXO3*) expression in a timely manner [25]. In response to heat shock, m6A modification on the 5′untranslated terminal region (UTR) of heat shock protein family H member 1 (*HSPH1*) can be protected by the reader YTH domain containing 1 (YTHDC1) to initiate cap-independent translation; m6A modification in GGAAU of lncRNA HSATIII can be sequestered by the reader YTHDC1 to repress m6A-dependent splicing [26–28]. Before adaptive immune initiation, loss of m6A modification in mRNAs of suppressor of cytokine signalling (SOCS) family genes stabilizes the mRNA and enhances the protein expression of targets, finally maintains the survival of naïve T cells [29]. Meanwhile, accumulated evidence shows that dysregulation of m6A levels and m6A regulatory proteins are also closely correlated with the progression of multiple tumours [26, 30–33]. As the first discovered methyltransferase, methyltransferase-like 3 (METTL3) accelerates growth of acute myeloid leukaemia (AML) cells via increasing m6A modification within the leukaemia-associated mRNA transcript presenting CCAAT enhancer binding protein zeta (CEBPZ) protein at transcriptional starting sites [34]. Likewise, in most OC cases, METTL3 plays as an oncogene by boosting invasion, migration and proliferation of OC cells, during which METTL3 targets different types of RNA, including pri-miRNA 126-5p, lncRNA RHPN1 antisense RNA 1 (head to head) (RHPN1-AS1) and mRNA AXL receptor tyrosine kinase (*AXL*) [35–37]. alkB homologue 5, RNA demethylase (ALKBH5), an m6A demethylase, can orchestrate m6A levels in the 3′UTR of programmed cell death-1 ligand-1 (*PD-L1*) mRNA, stabilize PD-L1 expression in intrahepatic cholangiocarcinoma (ICC), thus sensitize tumour cells to anti-PD1 immunotherapy [38]. There is also evidence for drug resistance modulation by ALKBH5 in OC [39]. Nie reported that upregulated ALKBH5 in OC decodes the m6A modification of Janus kinase 2 (*JAK2*) and stabilizes *JAK2* mRNA, subsequently contributing to cisplatin resistance, which is part of the standard adjuvant chemotherapy regimen [41]. Moreover, a recent study reported that YTH N6-methyladenosine RNA binding protein 1 (YTHDF1), an m6A ‘reader’, enhanced the overall translational output to fuel OC tumorigenesis and metastasis by recognizing m6A-modified eukaryotic translation initiation factor 3 subunit C (*EIF3C*) mRNA [40]. Collectively, numerous studies have underscored the importance of m6A signalling cascades in distinct cancer types, including OC.

This review will focus on emerging roles of m6A modification in OC. With this summary of new insights, this review not only improves our understanding of m6A signalling cascades but also provides potential markers for early screening for OC and relapse prediction, thus shedding light upon new strategies to target OC.

## Molecular basis for m6A modification

m6A functions throughout the entire process of RNA metabolism, which includes transport, translation, splicing and transcription, resulting in RNA stability and degradation [41, 42]. In global cellular RNAs, m6A nearly modifies 50% of the total methylated ribonucleotide [43, 44]. m6A modification ultimately converges on m6A-related regulators, including ‘writers’ (methyltransferases), ‘erasers’ (demethylases) and ‘readers’, which recognize substrates and show a clear preference for RRACH sequences (R  =  A or G, and H  =  A or C or U) [45, 46]. m6A mainly deposites on the 3′ untranslated region (3′UTR) and within the internal long exon of mRNA [47]. The 5′UTR m6A has also been observed and has been linked to selective eIF3-dependent and eIF4E-independent translation [48].

The writers include WTAP (Wilms tumour 1-associated protein), METTL3, METTL14 (methyltransferase-like 14), METTL16 (methyltransferase-like 16), RBM15 (RNA binding motif protein 15), KIAA1429 (vir-like m6A methyltransferase associated, VIRMA) and ZC3H13 (zinc finger CCCH domain-containing protein 13) and catalyse the formation of m6A [49]. The METTL3-METTL14 heterodimer takes part in most m6A modifications [50, 51]. Although it possesses no enzymatic methylation activity, WTAP is necessary for the WTAP-METTL3-METTL14 complex to function during the methylation process [52]. Each subunit of this complex possesses completely different catalytic activities and has very distinct roles during the methylation process. METTL3 exerts catalytic activity via adopting methyl group from S-adenosylmethionine (SAM), METTL14 mainly accounts for substrate recognition, and WTAP assists in directing the complex to nuclear spots [52–54].

m6A-modified mRNA is erased by ALKBH5 or fat mass and obesity-associated protein (FTO), yielding decreased total m6A levels in multiple cells [49, 55, 56]. ALKBH5 and FTO, members of the ALKB family, use Fe^2+^ and 2-oxoglutarate (2OG) as cofactors to decode the m6A modification [55, 57, 58]. Although several studies have unveiled the ability of FTO to erase N6,2′-O-dimethyladenosine (m6Am), most substrate of FTO is still the m6A modification, as the total amount of m6A is dominant in divergent cells [59–61]. FTO is extensively expressed in human different kinds of tissues, indicating its pivotal role in energy metabolism [62]. The indispensable interaction between ALKBH5 and DDX3, the latter of which belongs to the family of DEAD-box RNA helicases, may further dictate the essential role of ALKBH5 in RNA metabolism [63].

Currently identified m6A ‘readers’ include YTH domain family members (YTHDC1, YTHDC2, YTHDF1, YTHDF2 and YTHDF3), heterogeneous nuclear ribonucleoprotein (HNRNP) family members (HNRNPC and HNRNPA2B1), insulin-like growth factor 2 mRNA-binding proteins (IGF2BP1, IGF2BP2 and IGF2BP3), fragile X mental retardation 1 (FMR1) and leucine rich pentatricopeptide repeat containing (LRPPRC) [48, 64]. These ‘readers’ possess the ability to recognize m6A modifications in RNAs and generate functional signals [65]. For example, YTHDFs were shown to influence the stability and translation of mRNA [66–69]. YTHDC1 partially mediates splicing events [70–72]. YTHDF1 interacts with m6A-modified mRNA to enhance translation [69, 73, 74]. Through recruiting carbon catabolite repression 4-negative on TATA-less deadenylase complex (CCR4-NOT), YTHDF2 destabilizes and further decays the target mRNA [68, 75]. Taking the diverse types of RNAs into consideration, many more efforts have been made to discover m6A readers and unravel the underlying mechanisms.

## Biological functions of m6A related regulators in ovarian cancer

**Fig. 1:**
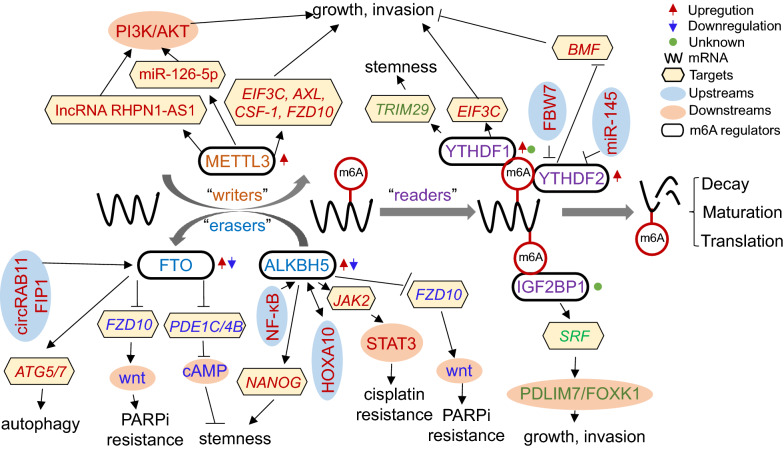
RNA-based m6A modifications play a vital role in OC progression. m6A regulators, which are closely correlated with OC progression, mainly include METTL3, FTO, ALKBH5, IGF2BP1, YTHDF1 and YTHDF2. FBW7 and miR-145 decrease the YTHDF2 protein level, leading to OC suppression. circRAB11FIP1-mediated FTO transcription can promote autophagy via m6A demethylation of ATG5 and ATG7. NF-κB stimulation upregulates ALKBH5 to induce demethylation of NANOG, which can promote tumour stemness. In an m6A-dependent manner, m6A regulators modulate the RNA maturation, stability and translation of miR-126-5p, lncRNA RHPN1-AS1, *JAK2*, *NANOG*, *FZD*, *PDE1C*/*PDE4B*, *SRF*, *TRIM29*, *EIF3C* and *BMF* in OC progression. Moreover, in a catalytic-independent manner, METTL3 promotes AXL translation to promote EMT

Similar to other tumours, the levels of RNA m6A modification are also dynamically modulated by these three regulator types in OC. Abnormal expression of m6A regulators predicts poor prognosis of OC patients and is involved in proliferation, invasion, metastasis and resistance via m6A-dependent and m6A-independent activity in OC [37, 39, 40, 76–82]. Figure 1 shows the pathological roles and underlying mechanisms of m6A regulators in OC and will be discussed below.

### m6A methylation ‘writers’

Although, at present, seven methyltransferases have been discovered, and all OC studies referring to m6A modification research mainly focus on METTL3 [35–37, 76, 83]. Accumulating evidence has revealed that METTL3 overexpression is extensively observed in OC tissues and predicts dismal prognosis. Both in vitro models and in vivo models, loss- and gain-of-function experiments have highlighted the importance of METTL3 during OC progression [35–37, 76, 83] (Table 1). In most other cancer types, METTL3 functions as an oncogene, including AML, breast cancer, colorectal cancer, gastric cancer, liver cancer, lung cancer, pancreatic cancer and prostate cancer. In several studies, METTL3 has also been found to possess the ability to suppress tumour progression, demonstrated by the higher expression of METTL3 in non-tumour tissues (vs. cancerous tissues) of renal cell carcinoma (RCC) and by the stronger proliferation in mutant METTL3 (vs. wild METTL3) of bladder cancer cells [84–86]. Researchers have identified several types of RNA as targets of METTL3 in OC, including pri-miRNA 126-5p, lncRNA RHPN1-AS1 and mRNA *AXL*, except rRNA and U6 snRNA, which is consistent with previous data indicating that METTL3 is not sensitive to any RNA structural context in vitro [46, 87, 88]. Scientists have also underscored the catalytic-independent activities of METTL3 that contribute to epithelial-mesenchymal transition (EMT) process in OC. A similar functioning mode was found in lung cancer cells demonstrating that METTL3 could recruit translation initiation factors (for example eIF3h) to augment the translation of oncogenes (for example *TAZ*), independent of its catalytic activity [89, 90]. However, what stimulates METTL3 overexpression in OC is still unclear, and there is a lack of experimental data inferring the interaction mode between METTL3 and the tumour microenvironment.

**Table 1:** The functions of m6A methyltransferases in OC

Writer	Expression	Function	Mechanism	Model	Ref.
METTL3	Up	Promotion	METTL3 methylates pri-miR-126-5p to promote miR-126-5p maturation, leading to the activation of PTEN-mediated PI3K/Akt/mTOR pathway	In vitro; in vivo	[36]
METTL3	Up	Promotion	METTL3 increases m6A levels of lncRNA RHPN1-AS1 and contributes to its stability	In vitro	[35]
METTL3	Up	Promotion	METTL3 promotes the translation of AXL catalytic-independently	In vitro; in vivo	[37]
METTL3	Up	Promotion	Independently of METTl14 and WTAP, METTL3 enhances m6A modification in the mRNA of oncogenes in OC, including *AXL*, *CSF-1*, *EIF3C* and *FZD10*	In vitro	[76]
METTL3	Up	Promotion	METTL3 knockdown downregulates p-AKT and the downstream effector Cyclin D1	In vitro	[83]

In endometrioid EOC, using a dot plot, Ma et al. showed increased global m6A levels compared to levels in adjacent tissues [76]. Further immunohistochemistry (IHC), western blot (WB) and real-time quantitative polymerase chain reaction (qRT-PCR) data revealed outstanding expression of METTL3 among METTL3, METTL14 and WTAP. In other studies, IHC data also displayed higher levels of METTL3 in OC cancerous tissue compared with the para-cancerous tissue [37, 83]. Liang et al. and Hua et al. both demonstrated that METTL3 was significantly correlated with tumour grade and TNM status, which was consistent with the data in endometrioid EOC verified by Ma et al. [37, 76, 83]. In xenograft mouse models, Hua et al. demonstrated that stable transfection with METTL3 accelerated tumour growth in OVCR-3 cells and that shMETTL3 significantly decreased tumour growth in SK-OV-3 cells in mice [37]. Present data indicates that METTL3 is an oncogene in OC. These preclinical and clinical data for METTL3 not only shed light on the possibility of treating METTL3 as a new prognostic factor for OC progression but also have encouraged scientists to explore the underlying molecular mechanisms to develop new targeting strategies for OC treatment.

m6A modification retunes the destiny of RNA, including maturation and stability [91]. Bi et al. found METTL3 controlled the m6A level of pri-miR-126-5p to accelerate its maturation, which could activate PI3K/AKT/mTOR signalling [36]. In addition, METTL3 knockdown decreased products of *miR-126-5p*, and further rescued miR-126-5p-induced proliferation, migration, invasion, and apoptosis inhibition in SK-OV-3 cells [36]. Furthermore, in a xenograft model, silencing METTL3 slowed tumour growth, whereas blockade of phosphatase and tensin homolog (PTEN) counteracted the anticancer effects of shMETTL3 [36]. These data suggest that METTL3 can execute its oncogenic role by activating PTEN/PI3K/AKT signalling in OC. Similarly, Liang et al. reported that METTL3 knockdown impeded OC cells proliferation and invasion, due to interference with AKT signalling cascades [83]. Through RNA immunoprecipitation (RIP) analysis in HEY cells (an EOC cell line), a recent study reported that shMETTL3 resulted in impaired m6A modification in total RNAs including lncRNA RHPN1-AS1, accompanied by accelerated decay of lncRNA RHPN1-AS1, which ultimately promoted tumorigenesis and metastasis of ovarian cancer in vitro [35]. The preliminary results presented in that study not only underpin the protumour roles of METTL3 during OC progression, but also identify different types of RNA as its targets, highlighting an emerging strategy to target mRNA.

Stable complexes are the main gateway to epigenetic methylation machinery. In addition to the KIAA1429/WTAP complex, one known complex of m6A methyltransferases consists of WTAP, METTL3 and METTL14 [53, 64]. Although it forms a protein complex to coordinate with WTAP/METTL14 in m6A modification, METTL3 seems at be the core of this process in endometrioid EOC, stemming from the fact that knockdown of METTL3, but not WTAP/METTL14, decreased the m6A levels of associated targets (determined in vitro by RIP-qPCR) [76]. Consistent with these previous data, RIP and WB assays showed that WTAP and METTL14 failed to occupy the transcripts of associated genes and upregulate the levels of associated proteins in OC cells (including TOV-112D and CRL-11731D) [76]. These results are contradictory to the observations from Liu et al. that METTL3, METTL14 and WTAP knockdown in HeLa cells led to decreased m6A in RNA by ∼30%, ∼40% and ∼50%, respectively [87]. This may be explained by differences between cell lines and different tumours, which highlights the necessity of exploring the functions of m6A regulators in OC as well as in other tumours. Ma et al. partially verified that METTL3 mediated m6A modification of targets to exert an oncogenic role in endometrioid EOC [76]. In addition, by employing the SRAMP tool, m6A modification located at the 3′UTR region of targets was significantly associated with OC, which is the common target site of METTL3 [76]. Another study demonstrated that METTL3 stimulates AXL translation to promote EMT independent of its catalytic activity in OC [37]. In this work, WB analysis showed that the catalytic mutant METTL3 augmented AXL protein levels to the same levels observed with wild-type METTL3 in OVCR-3 cells, underscoring the catalytic-independent role of METTL3 in OC [37], which will yield the possibility to define METTL3 as a ‘moonlighting protein’ [92]. However, there lack in vivo models to distinguish between catalytic-dependent and catalytic-independent functions of METTL3, specifically the knock-in alleles mice model. Afterall, it becomes increasingly clear that catalytic-independent functions also endow METTL3 with oncogenic roles in OC in addition to its catalytic-dependent function. Overall, these studies demonstrate that it may not be sufficient to inhibit only the catalytic activity in future strategies to target METTL3.

### m6A methylation ‘erasers’

In relation to human noncancer research, FTO possesses the ability to promote fat formation and obesity, and ALKBH5 participates in spermatogenesis, trophoblast invasion and ossification [93, 94]. In OC, dysregulation of ALKBH5 and FTO takes part in proliferation, apoptosis, migration, drug resistance, cancer stem cell development and tumour autophagy, contributing to the relapse of OC patients [39, 77–79, 95] (Table 2). In other types of cancer, ALKBH5 and FTO also participate in many pathological and biological activities, referring to proliferation, apoptosis, migration, invasion and metastasis [93, 94]. To date, a key set of studies have shown controversial roles of ALKBH5 in OC, which have also been observed in other cancer studies. We surmise that downstream signalling cascades are responsible for this apparent paradox, which will be discussed in the next section. Researchers have also found single-nucleotide polymorphisms (SNPs) in *FTO* and *ALKBH5*, which may have important roles in breast cancer, melanoma, pancreatic cancer and endometrial cancer [96–99]. The state of SNPs of *FTO* and *ALKBH5* in OC has rarely been observed but may be of prognostic value and deserves further exploration.

**Table 2:** The functions of m6A demethylases in OC

Eraser	Expression	Function	Mechanism	Model	Ref.
ALKBH5	Up	Promotion	ALKBH5-HOXA10 loop-mediates ALKBH5 expression, leading to *JAK2* m6A demethylation and stabilization, thus OC resistance to cisplatin	In vitro; in vivo	[39]
ALKBH5	Up	Promotion	In OC cells co-cultured with M2 macrophages, TLR4-NF-κB-mediated ALKBH5 upregulation causes *NANOG* mRNA demethylation and stabilization	In vitro; in vivo	[79]
ALKBH5/FTO	Down	Inhibition	ALKBH5 and FTO downregulation elevates m6A-mediated stability of *FZD10* mRNA and causes PARPis resistance by activating Wnt/β-catenin signalling in *BRCA*-mutated OC cells	In vitro	[78]
FTO	Down	Inhibition	FTO suppression increases m6A methylation to stabilize *PDE1C* and *PDE4B* mRNA, thus hindering CSC-related cAMP signalling	In vitro; in vivo	[77]
FTO	Unknown	Unknown	circRAB11FIP1 promotes autophagy through FTO-mediated demethylation of *ATG5* and *ATG7*	In vitro	[95]

Platinum-based chemotherapy is currently the first-line regimen for OC. PARPis have been licenced for treatment of patients with *BRCA* mutations due to their substantial clinical benefits. Drug resistance to platinum and PARPis inevitably emerges, remaining a clinical hurdle because of unraveled underlying mechanisms. Nie et al. showed that ALKBH5 mRNA and protein expression upregulated in platinum-resistant EOC cells or samples, demonstrated by qRT–PCR, WB and IHC assays [39]. Moreover, CCK-8 and EdU assays indicated that ALKBH5 boosted proliferation and hindered apoptosis in A2780 and HO8910 cells (two EOC cell lines), which were confirmed in vivo through animal studies using xenograft mouse models [39]. Then, transcriptional analysis verified the presence of a homeobox A10 (HOXA10)-ALKBH5 loop involved in chemoresistance in EOC cells, which was confirmed by correlation analysis between ALKBH5 and HOXA10 expressions based on 483 EOCs in TCGA database [39]. Further RIP-Seq, RNA-seq, RIP-qPCR and relative luciferase assays identified *JAK2* as an m6A demethylation substrate of ALKBH5, which was supported by data that blockade of either ALKBH5 or HOXA10 can rescue the activation of JAK2/STAT3 pathway [39]. This work underpins the importance of ALKBH5 in chemoresistance in OC, which may be applied to OC prediction and development of new treatment strategies. Through RNA-seq data, Fukumoto and the group found the whole levels of m6A-modified mRNA were similar between PARPi-resistant and parental PEO1 cells [78]. By analysing differentially modified genes between PARPi-resistant and parental PEO1 cells, Fukumoto et al. identified frizzled class receptor 10 (*FZD10*) as the top gene with increased m6A modification due to downregulation of FTO and ALKBH5, which was demonstrated by MeRIP-qPCR, qRT–PCR and WB assays in PEO1 cells [78]. Further TCF/LEF dual luciferase reporter assays and dual HR and NHEJ reporter assays uncovered the downstream signalling pathway Wnt/β-catenin, which was further validated by the synergistic effects on PARPi-resistant tumours in vivo, revealing that combining Wnt inhibitors overcomes PARPi resistance in *BRCA*-mutant OC [78]. This study casts further light upon the importance of FTO and ALKBH5 in resistance to PARPis. However, specific roles of FTO and ALKBH5 may be dependent on the type of drugs used and the mutation burden of OC, which deserves further research.

The cancer stem cell (CSC) is a subgroup of cancer cells, featured by self-renewal and regeneration. Recent works have highlighted the important roles of CSCs in OC carcinogenesis, metastasis and resistance [100–102]. By comparing normal fallopian tube epithelium (FTE), OC tissues and normal ovary tissues, Huang and the group revealed that the mRNA level of FTO was significantly lower in ovarian tumours, which was further confirmed by analyses of TCGA Affymetrix Exon-array data (569 high-grade serous and 8 control FTE specimens) [77]. Moreover, *FTO* levels were also significantly lower in the ALDH^+^ cell population (a stem cell population) derived from OVCAR5 and COV362 cells [77]. Loss- and gain-of-function experiments established an inhibitory role of FTO in self-renewal ability, colony formation ability, spheroid formation ability in vitro, and also the tumour initiation capacity in vivo [77]. Further integrative RNA sequencing and m6A mapping analyses revealed that cAMP signalling may be essential in hampering the stemness mediated by FTO blockade in OC [77]. In another study, through qRT-PCR analysis, Jiang et al. found upregulated *ALKBH5* in ovarian cancerous tissues than in normal tissues and downregulated *ALKBH5* in OC cell lines than in normal cells [79]. Further CCK-8 and flow cytometry assays showed protumour effects of ALKBH5 in ovarian cancer cells [79]. However, when cocultured with the M1 macrophage or the M2 macrophage, OC cells were divergently affected by ALKBH5 [79]. Specifically, the protumour abilities of OC cells were enhanced by coculture with M2 macrophages and inhibited by coculture with M1 macrophages. Collectively, Jiang et al. showed that OC cells upregulated ALKBH5 in inflammatory microenvironment to induce demethylation of Nanog homeobox (*NANOG*), which promoted the stemness and carcinogenesis of tumour cells [79]. The functions of demethylases are contradictory, which may be attributed to the complicated tumour microenvironment. Huang et al. observed that FTO downregulation was associated with cultures enriched with CSCs. Jiang et al. found that the effects of ALKBH5 on OC cells are dependent on a specific inflammatory microenvironment. These findings have advanced spirited debates on the effects of m6A regulators on interactions between tumours and their microenvironments. Continued work is important to clarify the effects and to benefit future m6A targeting strategies.

As a catabolic process, autophagy recycles cellular metabolites, macromolecules or organelles via lysosomes to maintain cellular homoeostasis [103–106]. A very large body of literature has established that autophagy is an important player during cancer development referring to proliferation, metastasis and resistance [107–110]. By using Torin 1-induced autophagy and sequencing in SK-OV-3 cells, Zhang et al. discovered that circRAB11FIP1 can promote autophagy, demonstrated by autophagy flux models (3-methyladenine, bafilomycin and Earle’s Balanced Salt Solution) and autophagy readouts (the distribution of mRFP-GFP-LC3) in SK-OV-3 and A2780 cells [95]. Further RIP assays in SKOV3 cells revealed that circRAB11FIP1 could bind *FTO* mRNA through 14 bp, indicated by the greater enrichment of *FTO* mRNA in the circRAB11FIP1 WT group in SK-OV-3 than in the 14-bp mutant group [95]. This interaction subsequently enhanced the expression of FTO in A2780 and SK-OV-3, demonstrated by manipulations of circRAB11FIP1 expression in a qRT-PCR assay [95]. Interestingly, by employing multiple autophagy flux models and autophagy readouts, Wang et al. discovered that FTO promoted autophagy in 3T3-L1 cells. By conducting LC–MS/MS quantification, RIP-qPCR, WB, MeRIP-qPCR and firefly luciferase activity assays, the authors also found that FTO directly erased the m6A modification in mRNAs of *Atg5* and *Atg7* in 3T3-L1 cells, which was partially verified by Zhang et al. in human OC cells [95, 111]. Moreover, in a generated *fto*-AKO model, deletion of Fto markedly inhibited autophagy, indicated by an attenuated LC3-II:I ratio, elevated Sqstm1 levels, and reduced Atg5 and Atg7 protein and gene expression [95, 111]. These two studies highlight the vital role of FTO in autophagy. Considering that the functions of autophagy are highly context dependent, the specific role of FTO in cancer remains contradictory, despite the suppression of FTO in PARPi-resistant OC cells and CSC OC cells [77, 78, 103].

### m6A methylation ‘readers’

In OC studies, m6A modification research mainly focuses on YTH domain family members (YTHDF1 and YTHDF1) and insulin-like growth factor 2 mRNA-binding proteins (IGF2BP1) [40, 80–82, 112]. Through functional and experimental analyses of ovarian cancer, recent studies underscore the protumour role of m6A ‘readers’ in vitro and in vivo, with regard to tumour growth, invasion and CSC phenotype [40, 80–82, 112] (Table 3). More recent work suggests that IGF2BP1, YTHDF1 and YTHDF1 mainly function as oncogenes in multiple cancer types, including colorectal cancer, hepatocellular carcinoma (HCC), gastric cancer, lung cancer, AML, pancreatic cancer, OC, bladder cancer, prostate cancer and melanoma [73, 113]. However, in a few cases, IGF2BP1, YTHDF1 and YTHDF1 exert suppressive functions. IGF2BP1 hampers tumour cell growth and invasion in breast cancer [113]. YTHDF1 blocks the migration and growth of melanoma [73, 114]. YTHDF2 hinders the progression of melanoma as well as gastric cancer [73, 115, 116]. These key sets of observations reinforce the context-dependent roles of m6A readers in multiple cancer types. This phenomenon is partly attributed to divergent functions of m6A targets, which can also be verified in OC, as discussed below.

**Table 3:** The functions of m6A RNA binding proteins in OC

Reader	Expression	Function	Mechanism	Model	Ref.
IGF2BP1	Unknown	Promotion	In a 3′UTR- and m6A-dependent manner, IGF2BP1 promotes SRF expression to augment SRF-dependent transcription	In vitro	[81]
YTHDF1	Unknown	Promotion	YTHDF1 interacts with m6A-modified *TRIM29* to promote TRIM29 expression, contributing to CSC features in cisplatin-resistant OC cells	In vitro	[82]
YTHDF1	Up	Promotion	In an m6A-dependent manner, YTHDF1 binds to m6A-modified *EIF3C* mRNA and augments EIF3C translation	In vitro; in vivo	[40]
YTHDF2	Up	Promotion	YTHDF2, degraded by FBW7, can recognize m6A-modifed *BMF* mRNA and accelerate decay of the latter	In vitro; in vivo	[80]
YTHDF2	Up	Promotion	YTHDF2, repressed by miR-145, promotes OC progression by decreasing global mRNA m6A levels	In vitro	[112]

m6A methylation reading proteins can selectively bind modified products of m6A to affect the metabolism of mRNA. IGF2BP1 and YTHDF1 are implicated in OC progression by augmenting the translation of target mRNA [40, 81, 82]. A case in point is the previous discovery that IGF2BP1-serum response factor (*SRF*) mRNA association upregulates SRF expression, followed by its activation of oncogenic transcriptional function in HCC (Huh-7 cell line) and OC cells (ES-2 cell line) [81]. In this work, to identify effector networks of oncogenic IGF2BP1, the author employed RNA-seq after IGF2BP1 knockdown to monitor mRNA abundance and cross-linking immunoprecipitation high-throughput sequencing (CLIP-seq) studies to assess binding sites in Huh-7 and ES-2 cell lines [81]. Further gene set enrichment analysis predicted *SRF* mRNA as a target of IGF2BP1 in both Huh-7 and ES-2 cell lines, which was subsequently validated by IGF2BP1 knockout using CRISPR/Cas9 technology both in vitro and in vivo [81]. This regulation process was presumably m6A-dependent, as shown by the decrease in IGF2BP1-*SRF* mRNA association upon METTL3/14 depletion in cells [81]. Finally, the IGF2BP1-SRF network promoted cell growth and invasion, demonstrated by a spheroid assay in cells, which was then verified by Kaplan–Meier Plot (Kmplot) analysis [81]. This work indicates that IGF2BP1 exerts an oncogenic role by targeting *SRF* in OC. Similar to this discovery, Hao et al. reported that YTHDF1 was recruited to m6A-modified tripartite motif containing 29 (*TRIM29*) to upregulate TRIM29 expression, which empowered cisplatin-resistant OC cells (A2780/DDP and SK-OV-3/DDP) with a CSC phenotype [82]. Using SILAC-labelled biotin pulldown assays followed by quantitative mass spectrometry, Hao et al. identified YTHDF1 as a *TRIM29* mRNA-binding protein, which was further established through a RIP assay, luciferase assay, WB and RIP-qPCR assay in A2780/DDP and SK-OV-3/DDP cells [82]. Next, functional analyses (including colony formation, Transwell analysis and a spheroid formation assay) indicated that YTHDF1 knockdown significantly suppressed the CSC features of A2780/DDP and SK-OV-3/DDP cells but showed no effects on the parental cells, which was consistent with the rescue environment induced by TRIM29 overexpression [82]. This study suggests that YTHDF1 behaves as an oncogene in tumours by targeting *TRIM29* in cisplatin-resistant OC cells. In another similar study, by analysing several cohorts of OC patients in TCGA pan-cancer database and GEO datasets (GSE66957 and GSE54388), Liu et al. discovered that YTHDF1 was elevated in various cancer types, including ovarian cancer, which was further established by qRT-PCR analysis of human fresh frozen ovarian tissues (n  = 35 for cancerous; n  = 12 for normal) and by IHC data from an OC tissue microarray (n  = 134) [40]. Next, Kmplot survival and FIGO stage analyses revealed that the expression of YTHDF1 significantly correlated with the prognosis of OC patients [40]. Functional analyses, including CCK-8, EdU staining, colony formation and Transwell assays, showed that YTHDF1 knockdown suppressed cell growth and migration in A2780 and SK-OV-3 cells in vitro, which was validated in vivo in a xenograft mouse model and an orthotopic mouse model [40]. Multiomics analyses, including RNA-seq, RIP-seq, eCLIP-seq and CLIP-qPCR, identified *EIF3C* as a YTHDF1-binding transcript that regulates RNA translation, which was consistent with the unchanged RNA abundance of YTHDF1 targets in multiomics analyses and was also further confirmed by the mutation of YTHDF1 and rescue experiments [40]. This study revealed that the oncogene *EIF3C* is a target mRNA for the m6A reader YTHDF1 in OC. In a word, these studies indicate that IGF2BP1 and YTHDF1 exert their oncogenic roles through their targets in OC, which provides opportunities to develop new diagnostic markers and new strategies to target IGF2BP1 and YTHDF1 in OC.

Early and more recent literature underlines the critical role of m6A regulators in OC. Exploration of downstream signalling cascades of the m6A regulator may answer the question that how m6A regulators function during OC progression [40, 81, 82]. There is another question: why are m6A regulators aberrantly expressed in OC? Explorations of upstream m6A regulators may elucidate this question. Recently, Xu et al. identified YTHDF2 as a novel substrate for E3-ubiquitin ligase (F-box and WD repeat domain containing 7) FBW7 in OC, the latter of which is a tumour suppressor [80]. After degradation via the proteasome system, impaired YTHDF2 impinged on mRNA decay of the proapoptotic gene bcl2 modifying factor (*BMF*) in vitro and in vivo [80]. In this study, by analysing data from TCGA RNA-Seq database (n  = 1207), Xu et al. displayed that YTHDF2 was elevated in the ovarian cancerous tissue and closely associated with poor prognosis of OC patients. This is in line with results from OC tissue microarrays (n  = 115) analyses via qRT-PCR, IHC and Kmplot survival analyses [80]. In addition, both in vitro and in vivo, blockade of YTHDF2 suppressed the growth of OC cells, as shown by CCK-8 assays, colony formation assays and a mouse xenograft model [80]. Further mechanistic research identified FBW7 as the E3-ubiquitin ligase for YTHDF2 and identified *BMF* mRNA as the m6A-modified substrate in OC [80]. Another clue can also be found by looking at an upstream factor of YTHDF2, namely, miR-145, which can hinder the abilities of OC cells to proliferate and migrate [112]. In this study, by analysing the ovarian cancerous tissue (n  = 31) and the normal ovarian tissue (n  = 14), Li et al. showed that increments of YTHDF2 protein in the ovarian cancerous tissue was closely related to clinical stages [112]. Moreover, functional analyses via CCK8, Transwell and Annexin V-FITC/propidium iodide staining analyses revealed that YTHDF2 conferred the abilities to proliferate and migrate on OC cells (SK-OV-3 and 3AO cells) [112]. Further TargetScan prediction, luciferase reporter, WB and qRT–PCR analyses showed that *YTHDF2* may be a direct target of miR-145, whose protumour ability could be rescued by YTHDF2 overexpression in SK-OV-3 and 3AO cells [112]. This crosstalk also occurred in HCC, in which miR-145 can directly target 3′UTR in *YTHDF2* mRNA to block YTHDF2 expression [117]. These two studies indicate tumour-promoting effects of YTHDF2 and dissect the upstream signalling in OC, redefining m6A modification as a signalling hub orchestrating several important pathways, such as the ubiquitin–proteasome system and miRNA.

## The double-edged sword role of m6A modification in OC

Demethylase knockdown has the same effect as methylase knockdown on m6A modification, and vice versa, which has been confirmed in multiple rescue experiments. As proof of this fact, m6A in transcription factor EB (*TFEB*) mRNA is balanced by both ALKBH5 and METTL3 [118]. In fact, the same effects on m6A modification have opposite biological functions in lung cancer, breast cancer and AML [21, 119]. Generally, in lung cancer, FTO is elevated to promote proliferation and invasion. Specifically, FTO can erase the m6A in ubiquitin specific peptidase 7 (*USP7*) and myeloid zinc finger 1 (*MZF1*) mRNAs, resulting in USP7 and MZF1 overexpression, which function as oncogenes in lung cancer [120, 121]. However, Du et al. reported METTL3 rescued miR-338-5p-mediated growth and invasion of lung cancer cells [122]. Breast cancer also presents this paradox. Through METTL14 overexpression or ALKBH5 knockdown experiments, some researchers have found that increasing global m6A levels impede propagation of human breast cancer cells [123]. Other researchers have discovered that increased global m6A levels were present in breast cancer patients and that METTL3 knockdown enhances apoptosis by hindering m6A modification of the oncogene *Bcl-2* and its translation [124]. In AML, demethylases (FTO and ALKBH5) possess the ability to inhibit disease progression in specific situations, so do methyltransferases (METTL3, WTAP and METTL14) [125–129]. These findings reveal the functional inconsistency of m6A modification in different cancers, including lung cancer, breast cancer and AML.

This paradox is also observed during OC progression. Almost all experimental data show METTL3 contributes to OC progression by participating in multiple signalling pathways, including the *AKT*, *EIF3C*, *AXL*, *CSF-1* and *FZD10* pathways, predicting poor prognosis for OC patients [35–37, 76, 83]. Consistent with these discoveries regarding m6A levels, ALKBH5 and FTO were impaired to elevate m6A modification of targeted genes and accelerate tumour progression [77, 78]. In *BRCA*-mutant OC cells, ALKBH5 and FTO downregulation led to PARPi resistance through stabilization of *FZD10* mRNA [78]. In OC stem cells, FTO enhanced cAMP signalling to impede the stemness features of OC via reducing the stability of phosphodiesterase 1C (*PDE1C*) and phosphodiesterase 4B (*PDE4B*) mRNA [77]. Contradictory discoveries have revealed that ALKBH5 overexpression endows OC cells with the ability to resist cisplatin and maintain stemness via separate m6A demethylation of *JAK2* and *NANOG* [39, 79]. Altogether these studies further reflect the contradictory roles of m6A modification in OC.

These paradoxes have raised a new question as to why the same m6A modification has opposite biological functions in OC and other cancer types. Considering that m6A readers fulfil the functions of writers and erasers, researchers have surmised that m6A readers may point to opposite functions caused by the same m6A modification, which is supported by the opposite biological functions of the same eraser ALKBH5 in OC. We speculate that downstream signalling cascades also contribute to the ‘double-edged sword’ role of m6A modification in OC. One of the reasons stems from the different targets (*JAK2* and *FZD10*) of the demethylase ALKBH5 reported respectively by Nie et al. and Fukumoto et al. in OC [39, 78]. Although the two studies both focused on drug resistance, the different expressions of ALKBH5 were found by Nie et al. in cisplatin-resistant EOC cells and by Fukumoto et al. in *BRCA2*-mutated PARPi resistant OC cells, which displayed distinct targets of ALKBH5. Another salient example lies in glioblastoma; METTL3/14 knockdown can promote tumorigenesis for glioblastoma stem cell (GSC) by decreasing m6A modification in the mRNA of the targeted oncogene ADAM metallopeptidase domain 19 (*ADAM19*) [130]. Paradoxically, it has been reported that METTL3 is essential to GSC maintenance and radioresistance through upregulation of the targeted mRNA of the oncogene SRY-box transcription factor 2 (*SOX2*) [131]. Although the two studies both focused on GSC phenotype, Cui et al. and Visvanathan et al. both used primary GSCs cultured as 3D tumourspheres but from different patients, which directed METTL3 to different targets and opposite roles in glioblastoma. These two paired studies demonstrate that apparent paradoxical roles of m6A signaling may finally converge on the targets of m6A modification in OC. Another issue that sheds light on the ‘double-edged sword’ role of m6A during OC progression refers to context-dependent functions of downstream signalling, such as autophagy. Autophagy is a catabolic process in which the endoplasmic reticulum forms a double membrane, creating a phagophore to engulf cellular cargo that subsequently fuses with a lysosome to produce an autolysosome, finally resulting in degradation of the cargo [107]. Several researchers have reviewed the pleiotropic functions of autophagy and identified autophagy as a ‘Janus-faced’ player in cancer development [132], which may partially dictate paradoxical roles of m6A modification during OC progression.

In conclusion, as shown by present evidence, m6A modification exerts opposite functions during OC progression, which relies on contradictory effects from two aspects: m6A regulators and their downstream signalling cascades.

## Opportunities for application of m6A modification in OC

Among myriads of distinct epigenetic modifications, m6A modification is the most prevalent and has the potential to solve the clinical problems related to OC treatment. One application of m6A modification is early detection and prognosis prediction in OC patients. Another application of m6A modification is to develop m6A-targeted therapeutics for OC patients.

### Early detection and prognosis prediction in OC patients

Multiomics studies and bioinformatics analyses of OC have explored the landscape features and prognostic values of m6A regulators. By integrating multiple databases, including GEO, TIMER, ROC Plotter and Kmplot, a study by Wang indicated that *HNRNPC* could predict relapse for patients with paclitaxel treatment; decreased *YTHDC1* and increased *RBM15* expression could predict metastasis; and *RBM15B*, *ZC3H13*, *YTHDF1* and *IGF2BP1* were closely associated with the immunological characteristics of OC patients [133]. Fan et al. underscored the important role of a three-gene signature (*IGF2BP1*, *VIRMA* and *ZC3H13*) for prognosis prediction [134]. Another four-gene signature reported by Wei et al. included *HNRNPA2B1*, which played an antitumour role [135]. Despite its good performance for prognosis prediction in both the training set and test set, the same author previously reported that HNRNPA2B1 played a protumour role in OC [136]. This paradox is explained not only by the dual role of m6A regulators but also by the inconsistency between protein and mRNA levels, which was also confirmed by Fan et al. [134]. In the study reported by Fan et al., compared with the normal tissue, the mRNA as well as protein expression level of ZC3H13 upregulated in the cancerous tissue, whereas the protein levels of IGF2BP1 and VIRMA differed from their mRNA levels [134]. In a word, there is an urgent need to conduct much more experiments to apply m6A modification to early detection or prognosis prediction.

Taking the current experimental evidence into consideration, METTL3 may be a proper marker for early detection and prognosis prediction in OC patients. However, there is limited clinical evidence showing correlations between different histotypes of OC tissues and METTL3 expression. How METTL3 expression compares with CA-125 concentration, which is currently the tumour marker widely used for early detection and prognosis prediction for OC patients, is still unknown.

### Possible strategies to target m6A modification in OC

Despite the clinical success of inhibitors of DNA epigenetics, including methyltransferases, to date, there are no approved drugs to target m6A RNA modification.

Early studies on m6A regulator-based targeting strategies mainly focused on demethylases. ALKBH5 and FTO exert their function via interacting with their cofactors and substrates, which are blocked by most reported inhibitors of FTO or ALKBH5 (such as rhein, CHTB, N-CDPCB, FB23-2, CS1/2, meclofenamic acid and entacapone) [137]. By screening studies of approved drugs, Huang et al. and Peng et al. separately identified meclofenamic acid (a nonsteroidal anti-inflammatory drug) and entacapone (combines with levodopa to treat Parkinson) as originally discovered inhibitors of FTO [138, 139]. FB23-2, a derivative of meclofenamic acid, was further demonstrated to suppress the propagation of human AML in vitro as well as in vivo [140]. Peng et al. revealed that one target of FTO in gluconeogenesis and thermogenesis was the transcription factor forkhead box protein O1 (*FOXO1*) mRNA, which participated in BCR-ABL1-independent imatinib relapse [141]. Meclofenamic acid and entacapone separately entered clinical trials for recurrent metastasis (NCT02429570) and gastrointestinal stromal tumours combined with imatinib (NCT04006769), highlighting the possibility of targeting m6A regulators for advanced or relapsed cancer [137]. Radicicol inhibits FTO in a dose-dependent manner (IC50  =  16.04 μM in enzymatic assays), which could enhance TRAIL-induced apoptosis in OC cells [142, 143]. Although these results hint at the possibility that radicicol may have antitumour effects in OC cells under specific conditions, inhibitors of m6A methyltransferases and demethylases have not been studied in OC and deserve in-depth research.

There are also several studies contributing to targeting of METTL3, and inhibitors of other writers are extremely rare. Through virtual screenings, Selberg et al. first identified four compounds that activated the METTL3-14-WTAP complex and led to the greatest increase in m6A level (21.4 ± 12.9%) in cellular assays [144]. Using a cofactor mimicking approach, Bedi et al. also discovered 7 potential inhibitors of METTL3 in silico [145]. Compound 2 was considered to be the most potential, with IC50  =  8.7 μM in enzymatic assays [145]. No cellular data were obtained in Bedi’s work. To date, there are two different selective small molecule inhibitors of METTL3, namely, STM2457 and UZH1a, which were independently reported by Yankova et al. and Moroz-Omori et al. [146, 147]. When binding with METTL3, the two inhibitors both occupy the SAM-binding site and reorganize Lys 513 of METTL3, which partly contributes to their selectivity. Consistently, SAM and sinefungin, pan inhibitors of methyltransferases, do not possess a specific binding mode [148]. Although the activity of STM2457 and UZH1a in OC is still unknown, both STM2457 and UZH1a could dampen the activity of METTL3 in the AML cell line MOLM-13 [146, 147]. Moreover, the propagation of several AML cell lines from human or mouse was blocked by STM2457 [146]. The efficacy of STM2457 was evaluated in a PDX model as well as a primary murine model, both of which carried different cancer drivers in vivo [146]. STM2457 displayed promising antileukaemic effects due to impaired AML propagation, diminishment of stem cells and improved survival with STM2457 treatment. Similar to AML, recent studies have also identified METTL3 as an oncogene in OC, providing important insights for exploration of METTL3 inhibitors in OC, which deserves further investigations.

To develop m6A regulator-based inhibitors for OC, researchers may consider the important question of which targeting technology is applicable. Considering that m6A-based RNA methyltransferases and demethylases may have catalytic-independent roles in OC, especially METTL3, proteolysis targeting chimaera (PROTAC) may be a promising technology to degrade this type of m6A regulator, which is consistent with the advice of Zeng et al. [50]. The first oral PROTAC (NCT03888612 for ARV-110, NCT04072952 for ARV-471) entered phase I clinical trials for prostate and breast cancer treatment separately and displayed promising data, showing good safety and efficacy [149]. The antitumour effects of PROTACs in OC have been established to target PI3K in OVCAR8 cells in vitro as well as in vivo, highlighting the advantages of PROTACs [150]. These data open new possibilities for targeting m6A regulators using the PROTAC technology.

m6A modification ultimately fine tunes the destination of mRNA, shedding light upon targeting mRNA processing as an emerging anticancer strategy, which may ignore the catalytic-independent role of methylases in OC. Alternative splicing is a key step in mRNA processing and participates in tumour invasion, proliferation, metabolism and drug resistance [151–155]. Specifically, in OC, a splice variant of *BRCA1* encodes BRCA1-Δ11q, resulting in resistance to PARPis compared with full-length BRCA1 in PDX models, which could be solved by targeting splicing [156]. As previously summarized, m6A-modified *JAK2*, *FZD10* and *TRIM29* all contribute to resistance to PARPis and cisplatin. Considering m6A as a signalling to modulate splicing [41, 42, 157], these observations are reminiscent of the question of whether splicing abnormalities exist for these m6A-modified mRNAs or other mRNAs (in addition to BRCA1) in OC [156, 158]. Although several splicing modulators have entered phase I clinical trials for cancer treatments, as reviewed by Desterro (e.g., NCT02841540 and NCT03614728) [159], this question is still important to employ splicing modulating as a strategy to target m6A-modified mRNA in OC. Another strategy targeting mRNA processing is therapeutic oligonucleotides with chemical modifications that possess the ability to inhibit gene expression [160]. To date, four therapeutic oligonucleotides have entered the clinic, mainly approved for non-tumour diseases. Many antitumour oligonucleotides have also been developed and evaluated in clinical trials, including Apatorsen (NCT01454089), AZD9150 (NCT02549651 and NCT01563302), AZD5312 (NCT02144051), MIR155 (NCT02580552), Custirsen (NCT01578655 and NCT01630733) and EZN-4176 (NCT01337518). However, the study of therapeutic oligonucleotides in OC is still deficient. There are also obstacles to applying this technology to OC. For example, which m6A-modified mRNA should be chosen? How can m6A-modified mRNA be specifically targeted. By solving these problems, therapeutic oligonucleotides are likely to become a hot research topic for development of RNA-based therapeutics to treat OC.

## Conclusion

In summary, aberrant m6A levels in OC, modulated by m6A regulators, participate in OC progression and predict relapse for OC patients. Considering the insights recently acquired, we can conclude several points: below. First, METTL3 is essential to the methyltransferase complex and acts as a protumour role during OC progression through catalytic-dependent or catalytic-independent pathways. The functions and modes of other methyltransferase complexes in OC remain an enigma. Second, m6A modification regulators have pleiotropic context-dependent functions in OC, indicating contradictory roles of m6A regulators. The mechanisms underlying this apparent paradox need to be further explored and may involve upstream stimuli, downstream signalling cascades, cancer cells and the tumour microenvironment. Last, similar to protein posttranslational modifications, crosstalk exists among m6A modifications and other RNA modification forms, which may partly contribute to the complexity of OC progression.

Collectively, this review not only updates our knowledge of m6A modification in OC, highlighting the important roles of m6A regulators and the underlying mechanisms, but also provides a rationale for development of new diagnostic markers and therapeutic strategies based on m6A modifications and regulators in OC.

## Data Availability

Not applicable.

## Abbreviations

2OG: 2-Oxoglutarate
ADAM19: ADAM metallopeptidase domain 19
ALKBH5: AlkB homolog 5, RNA demethylase
AML: Acute myeloid leukaemia
AXL: AXL receptor tyrosine kinase
BMF: Bcl2 modifying factor
CCR4-NOT: Carbon catabolite repression 4-negative on TATA-less deadenylase complex
CEBPZ: CCAAT enhancer binding protein zeta
CLIP-seq: Cross-linking immunoprecipitation high-throughput sequencing
CSCs: Cancer stem cells
PDE1C: Phosphodiesterase 1C
EIF3C: Eukaryotic translation initiation factor 3 subunit C
EMT: Epithelial to mesenchymal transition
FBW7: F-box and WD repeat domain containing 7
FMR1: Fragile X mental retardation 1
FOXO3: Forkhead box O3
UTR: Untranslated terminal region
FTE: Fallopian tube epithelium
FTO: Fat mass and obesity-associated protein
FZD10: Frizzled class receptor 10
HNRNP: Heterogeneous nuclear ribonucleoprotein binding protein
HOXA10: Homeobox A10
HSPH1: Heat shock protein family H (Hsp110) member 1
ICC: Intrahepatic cholangiocarcinoma
IGF2BP2: Insulin like growth factor 2 mRNA binding protein 2
IHC: Immunohistochemistry
JAK2: Janus kinase 2
Kmplot: Kaplan–Meier Plot
KIAA1429/VIRMA: Vir like m6A methyltransferase associated
LRPPRC: Leucine rich pentatricopeptide repeat containing
m6A: N6-methyladenosine
m6Am: N6,2′-O-dimethyladenosine
METTL3: Methyltransferase-like 3
METTL14: Methyltransferase-like 14
METTL16: Methyltransferase-like 16
mTOR: Mammalian target of the rapamycin
MZF1: Myeloid zinc finger 1
NANOG: Nanog homeobox
OC: Ovarian cancer
PARPi: Inhibitors of poly (ADP-ribose) polymerase
PD-L1: Programmed cell death-1 ligand-1
PDE4B: Phosphodiesterase 4B
SRF: Serum response factor
PROTAC: Proteolysis targeting chimaera
PI3K: Phosphoinositide 3-kinase
AKT: AKT serine/threonine kinase 1
PTEN: Phosphatase and tensin homolog
qRT-PCR: Real-time quantitative polymerase chain reaction
RBM15: RNA binding motif protein 15
RCC: Renal cell carcinoma
RHPN1-AS1: RHPN1 antisense RNA 1 (head to head)
RIP: RNA immunoprecipitation
SAM: S-adenosylmethionine
SETDB1: SET domain bifurcated histone lysine methyltransferase 1
SNPs: Single-nucleotide polymorphisms
SOCSs: Suppressor of cytokine signalling family genes
SOX2: SRY-box transcription factor 2
STAT3: Signal transducer and activator of transcription 3
YTHDC1: YTH domain containing 1
TFEB: Transcription factor EB
USP7: Ubiquitin specific peptidase 7
TRIM29: Tripartite motif containing 29
WB: Western blot
WTAP: Wilms tumor 1-associated protein
YTHDF1: YTH N6-methyladenosine RNA binding protein 1
ZC3H13: Zinc finger CCCH domain-containing protein 13
